# Training opportunities and the increase in the number of nurses in home-visit nursing agencies in Japan: a panel data analysis

**DOI:** 10.1186/s12913-019-4225-8

**Published:** 2019-06-20

**Authors:** Noriko Morioka, Suguru Okubo, Yoshie Yumoto, Yasuko Ogata

**Affiliations:** 1 Graduate School of Health Care Sciences, Tokyo Medical and Dental University (TMDU), 1-5-45 Yushima, Bunkyo-ku, Tokyo, 113-8510 Japan; 2 Institute of Ars Vivendi, Ritsumeikan University, 56-1 Toji-in Kitamachi, Kita-ku, Kyoto, 603-8577 Japan

**Keywords:** Home Care Services, Home health agencies, Human resources development, Home visit nursing, Nurse training

## Abstract

**Background:**

A training opportunity in which ongoing education is encouraged is one of the determinants in recruiting and retaining nurses in home-visit nursing care agencies. We investigated the association between ensuring training opportunities through scheduled training programs and the change in the number of nurses in home-visit nursing agencies using nationwide panel data at the agency level.

**Methods:**

We used nationwide registry panel data of home-visit nursing agencies from 2012 to 2015 in Japan. To investigate the association between planning training programs and the change in the number of nurses in the following year, we conducted fixed-effect panel data regression analysis.

**Results:**

We identified 4760, 5160 and 5025 agencies in 2012, 2013, and 2014, respectively. Approximately 60–80% of the agencies planned training programs for all staff, both new and former, during the study period. The means and standard deviations of the percentage change in the number of full time equivalent (FTE) nurses in the following year were 4.2 (19.8), 5.7 (23.5), and 5.8 (25.1), respectively. Overall, we found no statistically significant association between scheduled training programs and the change in the number of FTE nurses in the following year. However, the associations varied by agency size. Results of analysis stratified by agency size suggested that the first and second quartile sized agencies (2.5–4.0 FTE nurses) with scheduled training programs for all employees were more likely to see a 9.0% (95% confidence interval [CI]: 4.5, 13.5) and 8.5% (95% CI: 2.4, 14.5) increase in the number of FTE nurses in the following year, respectively. Similarly, the first and second quartile sized agencies with scheduled training programs for new employees were more likely to see a 4.7% (95% CI: 2.1, 7.2) and 3.3% (95% CI: 0.4, 6.2) increase in the number of FTE nurses in the following year, respectively.

**Conclusions:**

Ensuring training opportunities through scheduled training programs for all staff, both new and former, in relatively small-sized home-visit nursing agencies might contribute to an increase in the number of nurses at each agency.

**Electronic supplementary material:**

The online version of this article (10.1186/s12913-019-4225-8) contains supplementary material, which is available to authorized users.

## Background

Despite an increase in the demand for home-care owing to aging populations, the shortage of nursing workforce in home-care settings is a major issue in many countries [1–5]. Japan is a super-aging society, where 26.6% of people are elderly, that is, aged 65 years or older [6]. With the increase in the elderly population and the shifting of health policy to an integrated community care system that enables citizens to continue living in a familiar environment [7], the demand for home-visit nursing has been increasing [8, 9]. A forecast project estimated that a workforce of roughly 52,000 to 63,000 home-visit nurses will be needed in 2020, compared with the 33,000-strong workforce in 2014 [10, 11]. In addition to the shortage of home-visit nurses, overall, the number of nurses in small sized home-visit nursing agencies is particularly concerning. The majority of home-visit nursing agencies in Japan are small sized with fewer than five full-time equivalent (FTE) nurses [9]. In smaller sized agencies, operations are likely to be inefficient, resulting in increased burden on staff [9]. Moreover, they tend to be unprofitable compared to large agencies [12]. They are also unable to provide end-of-life care because of their inability to provide home-visit nursing 24 h a day owing to the shortage of manpower [13]. The Japanese Nursing Association has promoted polices that secure more home-visit nurses and expand the agency size [9].

Retaining nurses in a home-visit agency is a complex issue that is influenced by agency characteristics such as agency size and type, job characteristics, and nurse’s individual factors [14, 15]. Among these factors, the Japanese Nursing Association assumed that ensuring the availability of training opportunities in an agency is a key modifiable factor [9]. Since continuing education for home-visit nursing agencies in Japan is not required, a large percentage of home-visit nurses have reported feeling that their knowledge is insufficient [16, 17]. Training opportunities at agencies are related to decreasing the burnout of nurses [18, 19], increasing nurses’ job satisfaction [20], and increasing nurses’ intention to remain with their agencies [15]. Training opportunities are attractive considerations for home-visit nurses when they decide upon their place of work [21]. However, the association between ensuring that there is access to training opportunities for employees and an increase in the number of nurses in home-visit nursing agencies remains unclear.

We, therefore, aimed to investigate the association between providing training opportunities through scheduled training programs for all employees, both new and former, and an increase in the number of nurses in the following year in home-visit nursing agencies by using nationwide administrative data. In addition, since the association between training opportunities and the number of nurses might vary by agency size, we also aimed to investigate the association stratified by agency size in the previous year.

### Home-visit nursing in Japan

In Japan, home-visit nursing services were introduced to health insurance in 1994. Since the Long-term Care Insurance (LTCI) was introduced in 2000, home-visit nursing services have also been covered by LTCI [22]. To use a home-visit nursing service, an “order for nursing services” by a physician is required. This is done through an interview with the individual and an assessment of his or her needs. The home-visit nursing services provided include assistance for activities of daily living (ADL, e.g., bathing and grooming), rehabilitative training, wound care (e.g., bedsores), and end-of-life care [22].

As of 2015, there were approximately 33,000 FTE home-visit nurses in 8000 agencies throughout Japan [22]. The agencies should obtain a certification by the prefectural government to be reimbursed by national health insurance and/or LTCI. The certification criteria stipulate that each agency have at least 2.5 FTE nurses. A Survey on the Actual Situation of Visiting Nursing in 2014 by the Japanese Nursing Association showed that the average age of home-visit nurses was 47 year and that they had over 20 years’ nursing experience [23, 24]. Over 70% of the respondents had careers as hospital and/or clinic nurse before working as home-visit nurses, and only 0.6% of the respondents began work as home-visit nurses immediately after graduation from nursing schools.

## Methods

### Design

We conducted a longitudinal analysis of an unbalanced panel of nationwide administrative data for home-visit nursing agencies in Japan.

### Data source

Data on home-visit nursing agencies covering the period from 2012 to 2015 were drawn from the Information Publication System for Long-term Care database [25]. We requested disclosure of these data to the Ministry of Health, Labour, and Welfare, based on the Act on Access to Information Held by Administrative Organs. Data included information such as ownership, location, opening hours, contents of services, number of clients, number of staff, and training opportunities. From 2012, the Long-Term Care Insurance Act (Article 115–35–44) [26] required all managers of long-term care service agencies, including home-visit nursing agencies, to report their status to local (prefecture) governments annually. To enable people to obtain adequate information on long-term care services providers, the Ministry of Health, Labour and Welfare discloses this information online [25]. We used unbalanced panel data, which included both operational home-visit nursing agencies and those that were forced to close due to bankruptcy, to avoid the effect of survival bias on our research findings.

### Sample

**Fig. 1:**
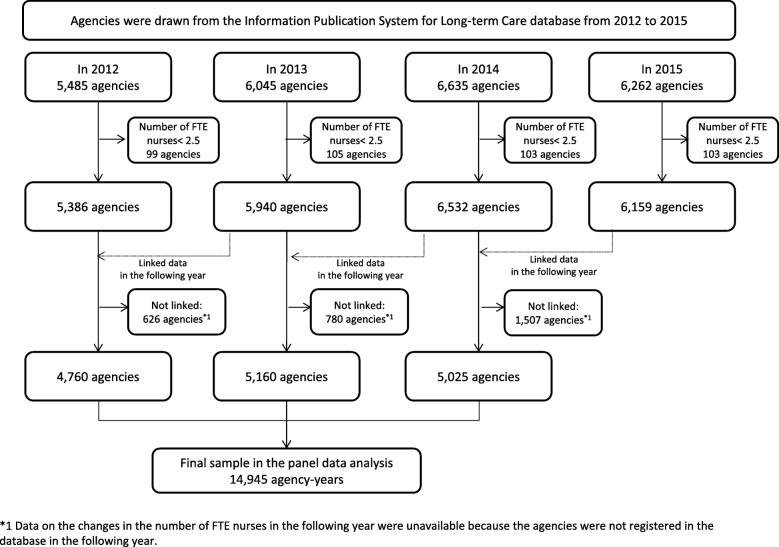
Flowchart of agency inclusion and exclusion criteria

We obtained data from 5485, 6045, 6635, and 6262 agencies that were registered in the database in 2012, 2013, 2014, and 2015, respectively. We excluded agencies that did not meet the inclusion criteria, which were as follows: the number of FTE nurses must meet the mandatory criteria of each agency by having at least 2.5 FTE nurses as certification criteria, and agencies must have existed in the database for two consecutive years because data on the changes in the number of FTE nurses in the following year must be available (Fig. 1). The 2015 data, therefore, were excluded from our panel data because we could not calculate the changes in the number of FTE nurses in the following year.

### Variables

#### Training opportunities

As a proxy for ensuring that training opportunities were available, we used the existence of scheduled programs for all employees, both new and former, as a variable by recording responses to the statement, “We have a scheduled program for training all employees and new employees,” with possible responses being yes = 1 (“with a scheduled training program”), and no = 0 (“without a scheduled training program”) in the database. We were unable to identify the contents of the training programs because the database we used in this study did not include such details.

#### Change in the number of FTE nurses

Percentage change in number of FTE nurses in the following year was calculated by the following equation:

*Percentage change in FTE nurses in the following year*_*it*_ = (*FTE nurses*_*i*(*t* + *1*)_ − *FTE nurse*_*i*(*t*)_)/*FTE nurses*_*i*(*t*)_ ∗ *100*.

In this equation, *i* denotes home-visit nursing agency and *t* denotes year.

#### Characteristics of home-visit nursing agencies

Other characteristics of home-visit nursing agencies were selected based on a review of the literature [14, 15, 17, 19, 20, 27] and available data. We used the number of FTE nurses as agency size because previous studies have suggested that the number of FTE nurses (called agency size) is related to nurses’ intention to stay at their agency [14] as well as the change in the number of nurses at a home-visit nursing agency after 16 months [17]. We also noted ownership of agencies (such as health-care corporation, for-profit corporation, and social welfare corporation) and the number of clients receiving home-visit nursing services by the agency during the month prior to the survey date. We calculated the number of clients per FTE nurse dividing the number of clients by the number of FTE nurses to show the nurses workload.

### Statistical analysis

We described the time trend in the number of FTE nurses of home-visit nursing agencies in terms of the proportion of those with scheduled training programs among the total number of agencies from 2012 through to 2014. To compare the number of FTE nurses between agencies with those without scheduled training programs at a cross-section, Mann–Whitney U tests were performed.

To investigate the association between scheduled training programs for all staff, both new and former, and the increasing number of FTE nurses in the following year, we performed a fixed effect (FE) (within agency) panel data regression analysis with unbalanced panel data for overall agencies. We also performed the models stratified by quartiles of agencies’ size (FTE nurses 2.5–3.0; 3.0–4.0; 4.0–5.6; and 5.6–50.8) to adjust for the agency size. The strength of FEs refers to the ability to control for confounding factors that vary across agencies but are constant over time [28]. In the database we used for this study, agency-specific variables such as administrators’ characteristics were lacking. However, those unobserved time-invariant agency-specific variables can be considered in the FE as fixed parts. Therefore, all models included control variables for all observed time-variant covariates (the FTE nurses, the squared of the FTE nurses, the number of clients per nurse), time FEs, and unobserved time-invariant agency-specific heterogeneity [28]. Owner agency type was excluded in the model due to time-invariant. We estimated the coefficient with robust standard errors. To examine the goodness of FE model fit compared with a random effect (RE) model or pooled ordinary least squared (OLS) model, an F test and a Hausman test were conducted [28]. Rejections of the F test and the Hausman test suggested that the FE model was better than both the RE model and pooled OLS. We checked whether our data met the assumptions of the FE panel data regression model using visual methods for strict exogeneity. The idiosyncratic errors were uncorrelated visually and numerical tests such as variance inflation factors (< 5) were conducted to check for multicollinearity [28]. *P* < 0.05 was considered to be statistically significant. All statistical analyses were performed using Stata (StataCorp), version 13.1.

## Results

**Fig. 2:**
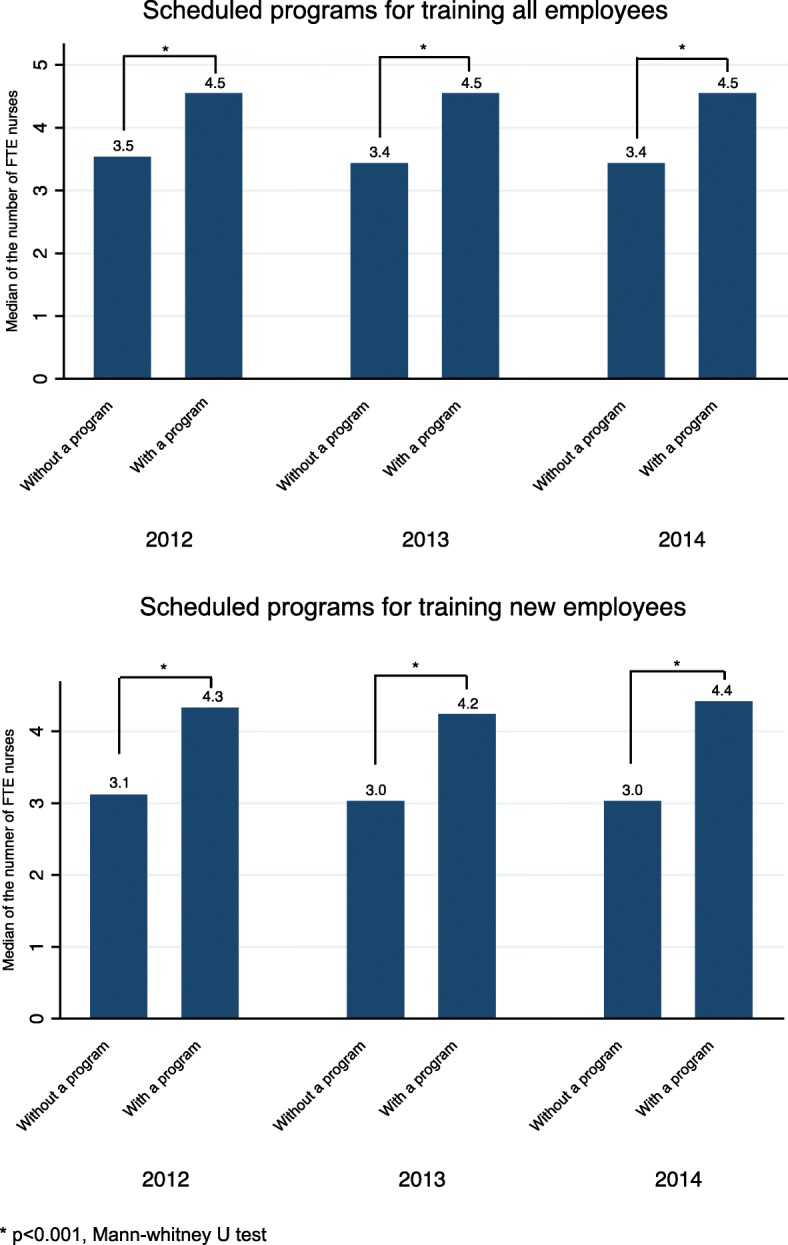
Comparison of agency size between agencies with and without scheduled training programs

We identified 4760, 5160, and 5025 agencies from 2012, 2013, and 2014, respectively for a total of 14,945 agency-years. Figure 1 shows the flow of agency selection. Table 1 shows that the proportions of agencies with scheduled training programs for all staff were 82.7, 82.2, and 80.4% for 2012, 2013, and 2014, respectively. For new staff, those proportions were 62.0, 61.8, and 61.4%, respectively. The means (standard deviations [SDs]) of the percentage change in the FTE nurses in the following year in 2012, 2013, and 2014 were 4.2 (19.8), 5.7 (23.5), and 5.8 (25.1), respectively. The means (SDs) of the number of FTE nurses in 2012, 2013 and 2014 were 4.9 (2.6), 5.0 (2.7), and 5.2 (2.9), respectively. From the results of the cross-sectional analysis, agencies with scheduled training programs for all employees were more likely to be larger than agencies without scheduled training programs (Fig. 2).

**Table 1:** Characteristics of home-visit nursing agencies from 2012 to 2014

	2012		2013		2014	
	*N* = 4760		*N* = 5160		*N* = 5025	
	Mean/n	SD/%	Mean/n	SD/%	Mean/n	SD/%
Number of FTE nurses	4.8	2.5	4.8	2.6	5.0	2.7
Number of clients	53.4	45.8	53.7	47.1	54.7	50.1
Number of clients per FTE nurse	11.5	9.5	11.4	9.5	11.3	9.5
Agency ownership						
Healthcare corporation	1694	35.6	1747	33.9	1640	32.6
Profit corporation	1362	28.6	1643	31.8	1788	35.6
Social welfare corporation	1533	32.2	1550	30.0	1386	27.6
Others	171	3.6	220	4.3	211	4.2
With a scheduled program for training all employees (yes = 1)	3935	82.7	4239	82.2	4042	80.4
With a scheduled program for training new employees (yes = 1)	2949	62.0	3188	61.8	3083	61.4
Percentage change in the number of FTE nurses in the following year	4.2	19.8	5.7	23.5	5.8	25.1

*FTE* full-time equivalent, *SD* standard deviation

Overall, no statistically significant association was found between scheduled training programs and the change in the number of FTE nurses in the following year (Table 2). Table 2 also shows that in the overall models, the number of FTE nurses was significantly associated with the percentage change in the number of FTE nurses in the following year. That is, the percentage changes in the number of FTE nurses tended to first decrease to approximately 30, then increase.

**Table 2:** Fixed-effect regression model for the percentage change in number of FTE nurses

	Scheduled training programs									
	For all employees					For new employees				
	Coef.	SE	95% CI			Coef.	SE	95% CI		
Planning training programs for all employees lagged	2.1	1.4	−0.7	4.8		0.5	0.7	−0.8	1.8	
Number of FTE nurses lagged	−23.9	1.6	−27.0	−20.8	*	−23.9	1.6	− 27.0	−20.8	*
Number of FTE nurses lagged squared	0.4	0.1	0.2	0.6	*	0.4	0.1	0.2	0.6	*
Number of clients per FTE nurses lagged	0.7	0.2	0.4	1.0	*	0.7	0.2	0.4	1.0	*
Year dummy (reference 2012)										
in 2013	2.8	0.4	2.2	3.5	*	2.9	0.4	2.2	3.6	*
in 2014	6.0	0.5	5.0	6.9	*	6.1	0.5	5.1	7.0	*
Intercept	96.5	5.9	84.9	108.0	*	97.5	5.9	85.9	109.1	*

14,945 agency-years

*CI* confidence interval, *Coef* coefficient, *FTE* full-time equivalent, *SE* standard error

* *p* < 0.001

Table 3 shows the results of models stratified by quartiles of agencies’ size in the previous year. Agencies with 2.5–4.0 FTE nurses with scheduled training programs for all employees were significantly associated with a 9.0% (95% CI 4.5–13.5) and 8.5% (95% CI 2.4–14.5) increase, respectively, in the number of FTE nurses in the following year compared with those without scheduled training programs for all employees. However, no significant association was observed for agencies with over 4.0 FTE nurses (Table 3, Additional files 1, 2). Similarly, small and mid-sized agencies with scheduled training programs for new employees were significantly associated with a 4.7% (95% CI 2.1–7.2) and 3.3% (95% CI 0.4–6.2) increase, respectively, of FTE nurses in the following year compared with those without scheduled training programs for new employees. However, once again, no significant associations were found in agencies with over 4.0 FTE nurses (Table 3, Additional files 1, 2).

**Table 3:** Fixed-effect regression model stratified by agency size

	Scheduled training programs									
	For all employees					For new employees				
	Coef.	SE	95% CI			Coef.	SE	95% CI		
1st quartile (2.5–3.0)	9.0	2.3	4.5	13.5	***	4.7	1.3	2.1	7.2	***
2nd quartile (3.0–4.0)	8.5	3.1	2.4	14.5	**	3.3	1.5	0.4	6.2	*
3rd quartile (4.0–5.6)	0.9	3.0	−4.9	6.8		0.1	1.4	−2.5	2.8	
4th quartile (5.6–50.8)	− 2.1	3.5	−8.8	4.7		−0.4	1.0	−2.4	1.7	

Adjusted for number of FTE nurses, squared number of FTE nurses, number of clients per FTE nurse, and year dummy

*CI* confidence interval, *Coef* coefficient, *FTE* full-time equivalent, *SE* standard error

* *p* < 0.05; ** *p* < 0.01; *** *p* < 0.001

## Discussion

To the best of our knowledge, this is the largest sample size study examining the association between training opportunities (through scheduled training programs) at home-visit nursing agencies and the change in the number of nurses in the following year, after adjusting for agency-specific confounding variables. Overall, we found no statistically significant association between scheduled training programs and the change in the number of nurses in the following year. However, we found that the associations varied by agency size. Relatively small agencies (< 4.0 FTE nurses) with scheduled training programs for all employees saw a statistically significant increase in the number of FTE nurses in the following year. In this study, we could not identify the underlying mechanisms explaining the increased number of nurses in home-visit nursing agencies with scheduled training programs. However, this could be explained by the fact that nurses prefer a workplace with a fulfilling training and education environment. In general, nurse retention was significantly associated with the practice environment, as measured by the Practice Environment Scale of the Nursing Work Index (PES-NWI), where active staff development or continuing education programs exist for nurses [29–31]. Similar to that found in a hospital setting, ensuring continuing education and a training environment may increase nurses’ job satisfaction and lead to nurse retention at home-visit nursing agencies [15, 20]. Ensuring training and education opportunities is especially important for new employees. A previous study also suggested that agencies should consider organized mentoring programs that improve new nurses’ overall confidence because those types of practices are often isolated and without a great deal of peer support [32].

We also found that small-sized agencies were less likely to have scheduled training programs both for all staff and for new staff. This may be related to the financial status of the agencies, because agencies with FTE nurses < 5 are less likely to be profitable than large agencies [12]. A vicious cycle thus occurs, whereby relatively small-sized agencies sustain a poor training environment; nurses then tend to leave and/or the agencies become less likely to hire new employees owing to the poor training environment. Consequently, the agency size remains small. An adequate training environment in such agencies may contribute to the retention of nurses and to the recruitment of new employees. Recently, some prefectural and/or municipal governments have developed a subsidize system for wages of a substitute nursing workforce in small home-visit nursing agencies when nurses in those agencies are absent owing to training or education commitments. To prevent small-sized agencies from falling into the above-mentioned vicious cycle, policies enforcing an adequate training environment for all staff in small-sized home-visit nursing agencies should be promoted.

Conversely, the associations were not significant among large-sized agencies. Several explanations may account for this finding. First, a ceiling effect of agency size may exist, regardless of training environment. A questionnaire study suggested that large-sized agencies were more likely to decrease the size of their nursing staff in the following year [17]. Second, the marginal effect of training programs on the increase of the nursing workforce at home-visit nursing agencies might diminish. Because a previous study in Japan suggested that nurses in large home-visit agencies (> 9 FTE nurses) did not have significantly higher work engagement scores than those in small agencies owing to wide spans of control, more nursing staff under the control of one manager may lead to less timely supervisory support [33]. Similar to the limited work engagement observed among large agencies, the effect of training and education opportunities on nurse retention might be limited in large agencies because wider spans of control may make reflecting on individual needs for training and education difficult.

Surprisingly, we also found that the association between agency size in the previous year and the growth rate of the agency followed a downward convex curve, which is inconsistent with the finding of a previous study suggesting that a linear positive association exists between agency size and nurses’ intention to stay [14]. Approximately 30 FTE nurses can be considered the inflection point that determines whether an agency remains mid-sized or aims for larger-scale deployment. In this study, we were unable to specify the underlying mechanisms explaining the trend. Further qualitative studies to investigate the home-visit nurse agency’s intentions to scale up are necessary. When estimating the forecast of the nursing workforce in home-care settings, this quadratic association between agency size and change in number of FTE nurses, but not the linear association, should be taken into account.

There were some limitations to this study. First, we used administrative data that lacked details of training programs. As a result, possible confounders were agency characteristics (i.e., wages, retention strategies, leadership style, etc.), patient case mix, and nurse characteristics (i.e., socioeconomic status, education level, job satisfaction, autonomy and independence, relationships with patients, etc.). Further studies are necessary to identify the types of program contents that are effective in recruiting and retaining nurses considering these possible confounders.

Second, a social desirability bias may exist because the data was reported by managers of the agencies. However, the bias is limited by false reporting, if discovered, results in the agency having to cancel approval by prefectural governments, which should prevent any deliberate falsification. Third, there might be an attrition bias in this study using an unbalanced panel data. We, however, considered a model that takes attrition bias into consideration because the test rejected the null hypothesis of no sample selection. Fourth, we could not estimate the effect of time-invariant variables, such as ownership, culture, regional characteristics, and characteristics of a manager in the panel data regression analysis, because the database we used in this study did not include such data. Further research is necessary to investigate the causality between the characteristics of agencies and the increase in the number of nurses at home-visit nursing agencies.

## Conclusions

Overall, we found no statistically significant association between ensuring training opportunities by scheduled training programs and the change in the number of nurses in the following year. However, ensuring training opportunities by planning training programs for all staff, both new and former, in relatively small home-visit agencies was associated with an increase in the number of home-visit nurses. Ensuring training opportunities through scheduled training programs for all staff, both new and former, in relatively small-sized home-visit nursing agencies might contribute to an increase in the number of nurses at each agency.

## Additional files

Additional file 1: Full model for fixed effect panel regression model for the % change in number of FTE nurses and scheduled training programs for all employees. (XLSX 15 kb)

Additional file 2: Full model for fixed effect panel regression model for the % change in number of FTE nurses and scheduled training programs for new employees. (XLSX 15 kb)

## Data Availability

The datasets used and/or analysed during the current study available from the corresponding author on reasonable request.

## Abbreviations

ADL: Activities of daily living
FE: Fixed effect
FTE: Full-time-equivalent
LTCI: Long-term Care Insurance
OLS: Ordinary least squared
PES-NWI: Practice Environment Scale of the Nursing Work Index
RE: Random effect
SDs: Standard deviations
